# Contribution of socio-economic and demographic factors to the trend of adequate dietary diversity intake among children (6–23 months): evidence from a cross-sectional survey in India

**DOI:** 10.1186/s40795-022-00655-z

**Published:** 2022-12-27

**Authors:** Divya Bhati, Abhipsa Tripathy, Prem Shankar Mishra, Shobhit Srivastava

**Affiliations:** 1 Bruyere, Canada; 2 PG Department of Statistics, Utkal University, Vani Vihar, Odisha Bhubaneswar, 751004 India; 3 Population Research Centre, Institute for Social and Economic Change, Bengaluru, 560072 Karnataka India; 4 Department of Survey Research & Data Analytics, International Institute for Population Sciences, Mumbai, 400088 Maharashtra India

**Keywords:** Adequate Dietary Diversity Patterns, Children aged 6–23 months, Socio-economic inequality, Decomposition India

## Abstract

**Background:**

The present study aims to estimate the factors contributing to the change adequate diversified dietary intake (ADDI) from 2005–06 to 2015–16 among children aged 6–23 months in India.

**Methods:**

A cross-sectional study was conducted using a large representative survey data. Data from the National Family Health Survey 2005–06 and 2015–16 was used. The effective sample size for the present study was 14,422 and 74,132 children aged 6–23 months in 2005–06 and 2015–16, respectively.

The outcome variable was minimum adequate dietary diversity intake. Binary logistic regression was used to evaluate the factors associated with ADDI. Additionally, the Fairlie method of decomposition was used, which allows quantifying the total contribution of factors explaining the decadal change in the probability of ADDI among children aged 6–23 months in India.

**Results:**

There was a significant increase in ADDI from 2005–06 to 2015–16 (6.2%; *p* < 0.001). Additionally, compared to the 2005–06 years, children were more likely to have ADDI [AOR; 1.29, CI: 1.22–1.35] in 2015–16. Mother's education explained nearly one-fourth of the ADDI change among children. Further, the regional level contribution of 62.3% showed that the gap was widening across regions between the year 2005–06 and 2015–16 in ADDI among children. The child's age explained 5.2% with a positive sign that means it widened the gaps. Whereas the household wealth quintile negatively contributed and explained by -5.2%, that means between the years the gaps has reduced in ADDI among children aged 6–23 months.

**Conclusion:**

Our findings indicate that increasing awareness of the use of mass media and improving the education levels of mothers would be beneficial for adequate dietary diversity intake among children aged 6–23 months. Investments should support interventions to improve overall infant and young children feeding practices in India.

**Supplementary Information:**

The online version contains supplementary material available at 10.1186/s40795-022-00655-z.

## Background

The burden of malnutrition is characterised by the simultaneous occurrence of undernutrition along with overweight and obesity or diet-related non-communicable diseases within populations [1]. It affects primarily low and middle-income countries (LMICs), and the dynamics of this burden are quite influenced by socio-economic levels [1]. Over the few decades, due to policy interventions, there has been a decrement in undernourishment by at least ten percent; however, malnutrition and deficiency in necessary micronutrients remain a critical public health challenge, especially in developing countries [2]. The UN adopted a resolution in 2016 proclaiming “United Nations Decade of Action on Nutrition” from 2016 to 2025 through which it’ll contribute to accomplishing the Sustainable Development Goals of putting an end to all forms of malnutrition (SDG2) [3].

Furthermore, malnourishment takes a toll on children's health by weakening the immune system while making a healthy, balanced and diversified diet a requirement for physical and mental well-being [3]. The escalating graph of a pattern of westernisation, urbanisation and mechanization that can be seen in most countries around the globe are associated with changes in diet towards high fat, high energy-dense foods and a sedentary lifestyle [4, 5]. So nutrient requirements for the human physique are multifaceted, making diversification in food intake compulsory for nutritional security [6, 7] and inadequate dietary intake, in terms of portion and assortment, is an instantaneous cause of poor nutritional outcome [8]. In this aspect, Dietary Diversity Scores (DDSs) enable us to measure the diversity in diets quantitatively and thus acquired prominence due to their conglomeration with varieties of health and nutrition-related outcomes [9]. In particular, Chandrasekhar et al. found evidence that diet diversity of children is statistically associated with child stunting, wasting and underweight [10]. Similarly in the Indian context, DDSs have been used to gauge the nutritive assessment of children [10–12]. As suggested by Agrawal, S. et al., Adequately Diversified Dietary Intake (ADDI) is a proxy indicator of a child's nutritional aspect, and the score can be computed by analysing the food items consumed by the children [13]. Hatloy et al. show that food variety scores can give a fairly nice assessment of the nutritional adequacy of the diet [14].

Multiple varieties of diet are somehow associated with escalated consumption of fibre and vitamins [14], and on the other hand, it contributes to high-calorific food consumption [15]. For example, the household dietary diversity score (HDDS) is a measure of a household’s economic ability to access a variety of foods [16]. Furthermore, research has demonstrated that an increase in dietary diversity is associated with an increase in per capita consumption, increase in per capita caloric availability and thereby betterment of socio-economic status [14, 17]. HDDS is a qualitative measure of food consumption that considers access of households to a variety of food but not at the individual level [16, 18]. A positive association can be observed between dietary diversity and three stanchions used to describe food security, namely availability, access and utilization [19]. As the availability, accessibility and affordability to diverse diet increases score of adequate diverse diet also increases. Feeding practices of infants and young children in the first two years of life are vital in improving child survival. Hence diversified food supply for children of 6–23 months is important, whereas, at the global level, only less than twenty-five percent of them get recommended diversified diet [20] Various documentation from South Asian countries suggests that India has a considerably low proportion of children (15%) who met minimum dietary diversity (MDD), followed by Nepal (34%), Bangladesh (42%), while the highest proportion of MDD can be seen in Sri Lanka [21], which indicates South Asian countries are lagging behind in MDD. Needless to mention that the world is in progress to denouement the problem of hunger and child malnutrition but however it is not on course to attain the 2025 and 2030 targets of child stunting and low birth weight, and for exclusive breastfeeding only 2025 target may be expected to be achieved [22].

Evidence from village level study in eastern India shows that access to Public Distribution System (PDS) hands out to enhanced dietary diversity as the target population is better able to afford diverse food items [19]. Besides this, lessened diversity scores can be attributed to the low socio-economic status group in the form of Scheduled Castes/Scheduled Tribes (SC/ST) [19]. A study on complementary feeding practices among children aged 6–23 months found that the prevalence of introduction of solid and semi-solid food among infants is very high in southern India (61%) compared to Central and Northern regions (38%); likewise, MDD is also highest in South (33%) and lowest in Central region (12%) [23]. A cross-cohort comparison of LMICs shows that four countries occupy places along the continuum of nutrition transition [24]. A survey on infants aged 6–23 months from 42 countries shows sufficient evidence for a pattern of feeding infants following Bennett’s Law, as households belonging to higher wealth index, introduce more diverse foods to their children at younger ages [25]. Families with higher income levels and better access to resources tend to have more diverse diets. In addition to this, they have better access to health care facilities, influencing nutritional adequacy, which is an important aspect of household wealth and resources translating into better outcomes for children [26, 27]. The findings from an in-depth nutrition baseline survey in Madhya Pradesh highlights very little dietary diversity, with more than 80% of women and 77% of children having consumed less than the daily recommended food groups [28]. A study by Borooah, demonstrates a pro-boy bias in dietary diversity, especially among children aged up to 24 months who are born to mothers with no formal education [29]. Results from a population-based study on dietary diversity (DD) reveals that taking into account individual-level, socio-economic factors explained 35.6% of total variation attributed to child DD at the community level, and 24.8% of the total variation is attributed to state level [30]. The Indian government has launched various policies like ICDS (Integrated Child development Scheme), *Poshan Abhiyan*, Mid-day meal programme etc., to create awareness about the necessity of a diverse and adequate diet [31], but the recent factsheet of NFHS-5 (2019–20) shows that the nutritional status of children has improved only imperceptibly.

There was a dearth of literature focusing on change in the ADDI among children aged 6–23 months in India. Additionally, there was no evidence about what factors contributes to the change in ADDI among children aged 6–23 months in India. Therefore, the present study aims to estimate the factors contributing to the change in ADDI from 2005–06 to 2015–16 among children aged 6–23 months in India. The study hypothesized that there was no change in the ADDI among children aged 6–23 months from 2005–06 to 2015–16 in India. The purpose of the study is to understand the ADDI patterns among children aged 6–23 months between the periods of 2005–06 to 2015–16.

## Methods

### Data

For this study, the data was used from the National Family Health Survey 2005–06 and 2015–16, which is a nationally representative cross-sectional survey conducted under the stewardship of the Ministry of Health and Family Welfare, Government of India [31]. The survey provides information on population, health, nutrition, and various demographic indicators at households as well as individual level. With the use of a stratified two-stage sampling procedure, NFHS gave the estimates for India as a whole, as well as each state and union territory level [31]. The detailed methodology, with complete information on the survey design and data collection, was published in the survey report [31]. The effective sample size for the present study was 14,422 and 74,132 children aged 6–23 months for 2005–06 and 2015–16, respectively.

#### Reliability and validity of data

NFHS data has maintained the quality of data and it’s reliability and validity. The NFHS-4 sample is a stratified two-stage sample. The 2011 census served as the sampling frame for the selection of PSUs (primary sampling units). PSUs were villages in rural areas and Census Enumeration Blocks (CEBs) in urban areas. Within each rural stratum, villages were selected from the sampling frame with probability proportional to size (PPS). In every selected rural and urban PSU, a complete household mapping and listing operation was conducted prior to the main survey [31]. Selected PSUs with an estimated number of at least 300 households were segmented into segments of approximately 100–150 households. Two of the segments were randomly selected for the survey using systematic sampling with probability proportional to segment size. Therefore, an NFHS-4 cluster is either a PSU or a segment of a PSU. In the second stage, in every selected rural and urban cluster, 22 households were randomly selected with systematic sampling [31].

### Variable description

#### Outcome variable

The outcome variable was adequately diversified dietary intake (ADDI) among children aged 6–23 months in India. In the NFHS-4, mothers were asked to provide a 24-h recall of foods and food groups given to their children. Children who received foods from four or more of the following food groups were defined to receive minimum dietary diversity: juice; tinned powdered/fresh milk; formula milk; fortified baby food; soup/clear broth; other liquids; chicken, duck, or other birds; bread, noodles, other grains; potatoes, cassava, tubers; eggs; pumpkin, carrots, squash; dark green leafy vegetables; mangoes, papayas, Vit A fruits; any other fruits; liver, heart, other organ meat; fish, shellfish; beans, peas, or lentils; cheese, yogurt, other milk products; other solid/semi-solid food; any other meat; and yogurt.

These food items were categorized into seven food groups followed by the WHO IYCF guidelines [32]: (1) "dairy products" (comprised of formula milk OR tinned powdered/fresh milk; OR cheese, yogurt, other milk products OR yogurt); (2) "legumes and nuts" (comprised of beans, peas, or lentils); (3) " roots, grains, and tubers" (comprised of soup/clear broth OR bread, noodles, other grains OR fortified baby food OR potatoes, tubers, cassava,); (4) "flesh foods" (comprised of heart, liver, other organ meat OR shellfish, fish OR chicken, duck, or other birds); (5) vitamin A rich fruits and vegetables" (comprised of carrots, pumpkin, squash OR dark green leafy vegetables OR mangoes, papayas, Vitamin A fruits); (6) "eggs" (comprised of eggs) "; and (7) "other fruits and vegetables" (comprised of any other fruits). The child was considered to take ADDI if he consumed four or more of the seven groups [13].

#### Explanatory variable

The other explanatory variables were divided into three categories: 1. Mother's characteristics 2. Child characteristics, 3. Household characteristics.

#### Mother’s characteristics

The mother’s age was coded as 15–24, 25–34 and 35 + years. The mother's educational status was coded as not educated, primary, secondary and higher. Media exposure was coded as exposed if the respondent was either watching television or reading a newspaper or listening to the radio and otherwise not exposed.

#### Child characteristics

The child's age was coded as 6–11, 12–17 and 18–23 months. The sex of the child was coded as male and female. Birth order was coded as 1, 2, and 3 + .

#### Household characteristics

The main explanatory variable was wealth status which represents the socio-economic status of a particular household. The variable wealth status was created using the information given in the NFHS survey. Scores were assigned to households based on the amount and types of consumer items they own, which range from a television to a vehicle or bicycle, as well as home features such as bathroom facilities, drinking water supply, and flooring materials. Principal component analysis was used to calculate these scores (PCA). The national wealth quintiles are calculated by assigning a score to each typical (de jure) household member, rating each individual in the household population according to their score, and then dividing the distribution into five equal groups, each having 20% of the population. The wealth index was categorized as poorest, poorer, middle, richer and richest [31]. Religion was coded as Hindu, Muslim, Christian and others. Other’s included Sikh, Buddhist, and Jain etc. Caste was coded as Scheduled Tribe, Scheduled Caste, Other Backward Class and others [33]. The Scheduled Caste includes a group of people who are socially separated and financially/economically disadvantaged as a result of their low caste position in Hindu society. The Scheduled Castes (SCs) and Scheduled Tribes (STs) are among India’s most economically disadvantaged groups. The OBC are people who have been labelled as "educationally, economically, and socially backwards." The OBCs are considered lower castes in the old caste system, although they are not untouchables [34]. The place of residence was coded as urban and rural. Regions of India were coded as North, Central, East, North-East, West and South.

#### Statistical analysis

Descriptive analysis, along with bivariate analysis, was used to find the differences in ADDI by background variables in 2005–06 and 2015–16. For the comparison of the prevalence of ADDI from 2005–06 to 2015–16, the study used a proportion test. The test of the normality and multicollinearity was conducted as pre-regression analysis. The model fit indices were tested using chi-square which was *p* < 0.001. STATA 14 was used for analysis.

The test statistic for comparing two proportions is defined as:

$$Z=\frac{{\widehat p}_1-{\widehat p}_2}{\sqrt{p\dot\ast\left(1-p\dot\ast\right)\left(\frac1{n_1}+\frac1{n_2}\right)}}\sim N\left(0,1\right)$$

where $p\dot{*}=\frac{{n}_{1}{\widehat{p}}_{1}+{n}_{2}{p}_{2}}{{n}_{1}+{n}_{2}}$; ${\widehat{p}}_{1}$ and ${\widehat{p}}_{2}$ are respectively the proportions of ADDI in the two periods (2005–06 and 2015–16). Similarly, ${n}_{1}$ and ${n}_{2}$ are the respective sample sizes in the two rounds of surveys [35].

Further, logistic regression analysis [36] was used to carve out the significant factors contributing to ADDI among children aged 6–23 months in India. To identify the underlying causes of the decadal difference in the prevalence of ADDI, the technique of decomposition had been used, which is now a day the most common approach used to identify and quantify inter-group differences. That is to compute the group difference (2005–06 to 2015–16) in the prevalence of ADDI among children and to decompose these differences into the major contributing factors, Fairlie's decomposition method [37, 38]. The method is commonly attributed to Blinder (1973) and Oaxaca (1973) [38]. This technique, however, is not appropriate if the outcome variable is dichotomous, such as ADDI, which is coded 0 "no" and 1 "yes". Hence, we used the extension of the Blinder-Oaxaca technique that is Fairlie decomposition which is appropriate for binary models to decompose the decadal change in the prevalence of ADDI into contributions that can be attributed to different factors [37, 38].

$${Y}^{t1}-{Y}^{t2}=\left[\sum_{i=1}^{{N}^{t1}}\frac{F\left({X}_{i}^{t1}{\beta }^{t2}\right)}{{N}^{t1}}-\sum_{i=1}^{{N}^{t2}}\frac{F\left({X}_{i}^{t2}{\beta }^{t2}\right)}{{N}^{t2}}\right]+\left[\sum_{i=1}^{{N}^{t1}}\frac{F\left({X}_{i}^{t1}{\beta }^{t1}\right)}{{N}^{t1}}-\sum_{i=1}^{{N}^{t1}}\frac{F\left({X}_{i}^{t1}{\beta }^{t2}\right)}{{N}^{t1}}\right]$$

where Y is the dependent variable (ADDI) at time t1 (2005–06) and t2 (2015–16), ${N}^{J}$ is the sample size for time t, ${X}^{J}$ is the row vector of average values of the independent variable, and ${B}^{J}$ is the vector of coefficient estimates for time t. This method of decomposition allows us to measure the absolute contribution of factors explaining the decadal variation (2005–06 to 2015–16) in the probability of ADDI among children aged 6–23 months in India. Stata14 [39] was used to carry out the analysis. The authors used *svyset* in Stata 14 command to control the analysis for complex survey design of National Family Health Survey. Additionally, survey weights were used to make the estimates nationally representative.

### Patient and public involvement

No patient involved.

## Results

Table 1 presents the socio-economic and demographic characteristics of the study population by using two rounds of data 2005–06 & 2015–16 in India. During 2005–06, half of the mother's age group was in the 15–24 years (52.4%) as compared to 45.6% in the same age groups in 2015–16. In the mother's educational statuses between these two periods as in 2005–06, nearly 47% of women were illiterate compared to 27.4% in 2015–16. However, secondary (33.1% vs 47.4%) and higher education (6.1% vs 11.7%) levels have increased among women between the periods of 2005–06 and 2015–16. Among the child age groups, it was almost the same between the periods. However, the childbirth order has changed between the periods. During 2005–06, 32% of children were born as first-order among women as compared to 38.2% in 2015–16. Further, 40% of children were born the third and more order among women in 2005–06 as compared to 28.5% in 2015–16. Among the wealth quintiles, no change has been seen between these two periods. In the poorest quintile, one-fourth population is fallen into this category; however, 15% of the population is fallen into the richest quintile in both years. During 2005–06, 20.6% of the population belonged to Scheduled Caste (SC), 9.6% of Scheduled Tribe (ST) as compared to 21.8 & 10.4% in 2015–16 respectively. During 2005–06, one-fourth of the population were residing in urban areas as compared to 28% in 2015–16. Regional wise huge variations can also be found in the study population between two periods.

**Table 1:** Socio-economic and demographic characteristics of the study population in India

**Background characteristics**	**2005–06**			**2015–16**		
	**Sample**	**Percentage**	***P*-Value**	**Sample**	**Percentage**	***P*-value**
**Mother’s characteristic**						
**Mother's age (in years)**			< 0.001			< 0.001
15–24	7,563	52.4		33,810	45.6	
25–34	6,068	42.1		36,184	48.8	
35 +	791	5.5		4,137	5.6	
**Mother's educational status**			< 0.001			< 0.001
Not educated	6,769	46.9		20,306	27.4	
Primary	1,996	13.8		10,034	13.5	
Secondary	4,780	33.1		35,123	47.4	
Higher	877	6.1		8,669	11.7	
**Media exposure**			< 0.001			< 0.001
Exposed	4,471	31.0		19,244	26.0	
Not exposed	9,951	69.0		54,888	74.0	
**Child characteristic**						
**Child's age (in months)**			< 0.001			< 0.001
6–11	4,859	33.7		25,235	34.0	
12–17	4,794	33.2		24,455	33.0	
18–23	4,769	33.1		24,441	33.0	
**Sex**			< 0.001			< 0.001
Male	7,590	52.6		38,793	52.3	
Female	6,832	47.4		35,339	47.7	
**Birth order**			< 0.001			< 0.001
1	4,615	32.0		28,348	38.2	
2	4,018	27.9		24,642	33.2	
3 +	5,789	40.1		21,143	28.5	
**Household characteristics**						
**Wealth quintile**			< 0.001			< 0.001
Poorest	3,542	24.6		18,110	24.4	
Poorer	3,190	22.1		16,073	21.7	
Middle	2,794	19.4		15,145	20.4	
Richer	2,663	18.5		13,722	18.5	
Richest	2,234	15.5		11,081	15.0	
**Religion**			< 0.001			< 0.001
Hindu	11,235	77.9		58,331	78.7	
Muslim	2,460	17.1		12,213	16.5	
Christian	314	2.2		1,551	2.1	
Others	413	2.9		2,037	2.8	
**Caste**			< 0.001			< 0.001
Scheduled Caste	2,974	20.6		16,126	21.8	
Scheduled Tribe	1,380	9.6		7,739	10.4	
Other Backward Class	5,736	39.8		32,618	44.0	
Others	4,332	30.0		17,649	23.8	
**Place of residence**			< 0.001			< 0.001
Urban	3,713	25.8		20,812	28.1	
Rural	10,709	74.3		53,320	71.9	
**Regions**			< 0.001			< 0.001
North	1,878	13.0		9,701	13.1	
Central	1,661	11.5		19,561	26.4	
East	128	0.9		19,325	26.1	
North East	3,018	20.9		2,556	3.5	
West	5,880	40.8		9,458	12.8	
South	1,857	12.9		13,532	18.3	
**Total**	14,422	100.0		74,132	100.0	

Table 2 depicts the percentage distribution of changes in the adequate dietary diversity intake (ADDI) among children 6–23 months across different socio-economic and demographic groups of the two periods 2005–06 & 2015–16 in India. Overall, in 2005–06, only 14.2% of the children had ADDI compared to 20.4% in 2015–16, and the total difference was 6.2% between these two years in the ADDI among children that were significantly found in India. In the 15–24 age group of mothers, the ADDI differences were 5.6%, between the periods of 2005–06 (13.4%) & 2015–16 (18.9%) as the mother's age increased that has significantly led to more differences or widened in the ADDI among children. Similarly, the mother's educational status had a significant effect on ADDI among children between the periods. Increasing the mother's educational status has led to a decrease in the differences in the ADDI between the periods. Children belonged to the illiterate mother, during 2005–06, the ADDI was 7.8% compared to 14.7% in 2015–16. Whereas among the higher educated mother, it was 32.3% in 2005–06 compared to 28% in 2015–16. Media exposure has also made influenced the ADDI between these two periods (7.8% vs 14%). Child's age group also matters in the ADDI, as compared to age group 6–11, the age group of 18–23 were having more ADDI, and between the years it has increased 5.2 to 21% in 2005–06 and 9.6 to 28.6% in 2015–16. In the sex of the child, between the years, the changes were seen (for the male child: 14.4% vs 20.3%; and for the female child: 13.9% vs 20.6%). The birth order of children among women has significantly impacted ADDI.

**Table 2:** Differences in the percentage of ADDI among children aged 6–23 months by their background characteristics in India

Background characteristics	2005–06	2015–16	Difference	*p*-value
	%	%	%	
**Mother's characteristic**				
**Mother's age (in years)**				
15–24	13.4	18.9	5.6	< 0.001
25–34	15.7	21.9	6.2	0.035
35 +	10.2	20.0	9.9	0.007
**Mother's educational status**				
Not educated	7.8	14.7	6.9	< 0.001
Primary	14.8	17.4	2.6	0.045
Secondary	19.6	22.7	3.1	0.633
Higher	32.2	28.0	-4.2	< 0.001
**Media exposure**				
Exposed	7.8	14.0	6.2	< 0.001
Not exposed	17.1	22.7	5.6	0.004
**Child characteristic**				
**Child's age (in months)**				
6–11	5.2	9.6	4.4	< 0.001
12–17	16.5	23.4	7.0	< 0.001
18–23	21.0	28.6	7.6	0.036
**Sex**				
Male	14.4	20.3	5.9	0.002
Female	13.9	20.6	6.7	< 0.001
**Birth order**				
1	16.4	20.4	4.1	0.231
2	16.3	22.9	6.6	0.031
3 +	11.0	17.6	6.6	< 0.001
**Household characteristics**				
**Wealth quintile**				
Poorest	7.1	15.2	8.1	< 0.001
Poorer	11.1	18.2	7.1	< 0.001
Middle	13.7	20.9	7.2	< 0.001
Richer	18.5	25.4	6.9	< 0.001
Richest	25.3	25.5	0.2	< 0.001
**Religion**				
Hindu	13.9	19.7	5.8	< 0.001
Muslim	14.2	22.5	8.3	< 0.001
Christian	23.2	31.6	8.4	< 0.001
Others	13.7	20.7	7.0	0.032
**Caste**				
Scheduled Caste	12.4	19.5	7.2	0.004
Scheduled Tribe	9.3	19.2	9.9	< 0.001
Other Backward Class	13.1	19.9	6.8	0.011
Others	18.4	22.8	4.4	0.038
**Place of residence**				
Urban	20.0	25.7	5.7	< 0.001
Rural	12.2	18.4	6.2	< 0.001
**Regions**				
North	14.3	16.2	1.9	0.201
Central	28.5	12.1	-16.4	< 0.001
East	25.5	22.2	-3.3	0.135
North East	16.3	29.2	12.9	< 0.001
West	9.1	18.3	9.2	< 0.001
South	13.1	32.8	19.7	< 0.001
**Total**	14.2	20.4	6.2	< 0.001

Household characteristics have significantly impacted the ADDI among children aged 6–23 months, and the differences were found widely between the periods. During 2005–06, in the poorest households, only 7.1% of ADDI among children was there as compared to 15.2% in 2015–16. Whereas in the richest quintile, between these two periods, the ADDI was the same (25.3% vs 25.5%). The differences in ADDI among children were decreased between the periods with increasing the wealth quintiles. Among the religious groups, during 2005–06, Hindu (14%), Muslim (14%), Christian (23.2%) while 2015–16, it has increased across the religions as Hindu (20%), Muslim (19%) and Christian (31.6%). In the social group, during 2005–06, 12.4% of children who belonged to the SC category had ADDI compared to 19.5% in 2015–16. A similar pattern can also be seen across other social categories. The gaps across social groups revealed that SC & ST children were having low ADDI compared to OBC (Other backward Classes) and others. Place of residence has also been significantly associated with the relative change to ADDI among children between these two years. Further, regional variations were significantly found in the change of ADDI among children between 2005–06 to 2015–16 (Table 2).

Table S1 (supplementary file) represents the pooled logistic regression analysis to see the association of factors with ADDI among children aged 6–23 months in India. Compared to the 2005–06 years, children were more likely to have ADDI [AOR; 1.29, CI: 1.22–1.35] in 2015–16.

Table 3 presents the estimate of Fairlie decomposition for ADDI among children between the years 2005–06 and 2015–16. After controlling other factors, the predictive probability of ADDI among children in 2005–06 was 0.14 compared to 0.20 in 2015–16. Further, the results indicate that 86% of such differences are explained by the factors included in the analysis. The remaining unexplained was 14% which is due to the year change of 2005–06 to 2015–16.

**Table 3:** Fairlie decomposition estimates for ADDI among children aged 6–23 months in India

Number of observation	88,554
Number of observations (2005–06)	14,422
Number of observations (2015–16)	74,132
Predictive probability for food diversity among children aged 6–23 months in 2005–06 (P1)	0.14
Predictive probability for food diversity among children aged 6–23 months in 2015–16 (P2)	0.20
P1-P2	-0.06
Explained	-0.05
Total explained	86%

Table 4 represents the detailed Fairlie decomposition analysis to quantify the contribution of different socio-economic and demographic determinants explaining the gap in the ADDI among children aged 6–23 months between these two periods. A positive contribution indicates that the particular variable has made an impact on the gap in the ADDI among children between the two periods. At the same time, the negative contribution indicates that it has reduced the gap between the years in ADDI among children. The regional levels contribution of 62.3% showed that the gap was widening across regions between the year 2005–06 and 2015–16 in ADDI among children. Highest ADDI among children can be found in central and eastern regions of India. Further, the mother's education significantly explained nearly one-fourth of the ADDI among children. The child's age explained 5.2% with a positive sign that means it has made an impact on widening the gaps in ADDI among children between the periods. Whereas the household wealth quintile negatively contributed and explained by -5.2%, that means between the years; the gaps have reduced in ADDI among children aged 6–23 months. Over the period, the household wealth quintile-related inequality has reduced and it made a significant impact on dietary diversity among children. The caste variable did not make any such changes between the periods. Further, there were also contributing factors such as place of residence, mother's age and mass media that were positively related and widened the gaps between the years in ADDI. However, there are other contributing factors like birth order of child (-1.7%), Caste (-0.8%), and religion (-0.2%) that showed a negative association and have reduced the gaps between the years in ADDI among children aged 6–23 months. Between the two periods, the change in ADDI was found at 0.06%. The results finally revealed that the highest contribution was from household characteristics, followed by mother’s characteristics and child’s characteristics.

**Table 4:** Fairlie decomposition analysis estimates for ADDI among children aged 6–23 months by their background characteristics in India (2005–06 to 2015–16)

Background characteristics	Coefficients	*p*-value	Percentage contribution	
**Mother's characteristics**				
**Mother's age (in years)**				
15–24				0.8
25–34	-0.001	0.412	0.9	
35 +	0.000	0.740	-0.1	
**Mother's educational status**				
Not educated				24.8
Primary	0.001	0.048	-1.4	
Secondary	-0.007	< 0.001	10.5	
Higher	-0.010	< 0.001	15.8	
**Media exposure**				
Exposed				0.6
Not exposed	0.000	0.383	0.6	
**Child characteristics**				
**Child's age (in months)**				
6–11				5.2
12–17	0.002	0.426	-2.4	
18–23	-0.005	0.022	7.6	
**Sex**				
Male				0
Female	0.000	0.849	0.0	
**Birth order**				
1				-1.7
2	0.000	0.579	0.3	
3 +	0.001	0.223	-2.0	
**Household characteristics**				
**Wealth quintile**				
Poorest				-5.2
Poorer	0.000	0.517	0.6	
Middle	0.000	0.818	0.2	
Richer	0.001	0.288	-1.7	
Richest	0.003	0.009	-4.3	
**Religion**				
Hindu				-0.2
Muslim	0.000	0.755	0.0	
Christian	0.000	0.212	-0.2	
Others	0.000	0.500	0.1	
**Caste**				
Scheduled Caste				-0.8
Scheduled Tribe	0.000	0.809	-0.1	
Other Backward Class	0.000	0.812	0.3	
Others	0.001	0.564	-1.0	
**Place of residence**				
Urban				0.2
Rural	0.000	0.647	0.2	
**Regions**				
North				
Central	-0.019	< 0.001	30.7	62.3
East	-0.022	< 0.001	34.5	
North East	0.006	0.007	-10.2	
West	-0.005	0.054	8.5	
South	0.001	0.154	-1.2	
**Total contribution**			86.1	

## Discussion

Many researchers have studied malnutrition in children in low and middle-income groups of countries [40–42]. This study carries important policy recommendations for healthcare researchers, governance, and public health professionals, as it presents evidences on the socio-economic predictors of adequately diversified dietary intake (ADDI) among children aged 6–23 months at maternal, children, and household levels. Good health and essential nutrition intake contribute to the consumption of diversified food items [26]. Including adequate food for younger children are effective strategies for their growth and development [43]. In resource-constrained countries, the government has recognized the lack of sufficient and diverse dietary patterns as a healthcare challenge [44]. With the aim to find challenges, the study shows the transition in ADDI from 2005–06 to 2015–16 and determined predictors of the socio-economic inequality in ADDI among children aged 6–23 months. The total difference was 6.3% between these two years; however, only 20.4% of the children had ADDI in 2015–16. It also brings out the need to design age-appropriate strategies to increase complementary diversified dietary intake among children.

In the study, the mother's age was associated significantly with a child's ADDI. Mothers whose age belongs to the 25–34 years and higher age groups led to widened dietary diversity than mothers that belong to the 15–24 age group. The intake of a diverse and nutritious diet may be attributed to the increase in the mother's experience and knowledge in child feeding with age. Similar findings were reported in the low income country like consistent Ethiopia [45]. This study also revealed a 5.6% increase in ADDI in 2015–16. In both periods, the mother’s education above the primary level was the significant predictor of ADDI. A mother’s education has several potential roles and implications for children. Mother’s education and even empowerment serve as a foundational path to improving child’s nutrition and health. Educated mothers may have confidence and feel empowered to understand the appropriateness of nutritious food for their children's growth and provide adequate diverse dietary food. This finding corroborates with other studies [33, 46]. A review of multiple studies showed a positive correlation between a mother’s ability to take decisions and empowerment with children’s growth and development [47]. The review also suggests the literature is limited in the context of a child’s nutrition and maternal autonomy hence, gives directions for future research.

In this study, mothers who had exposure to mass media were found to be significantly associated with ADDI between these two periods (7.8% vs 14%). Previous studies have also documented the role of media in attaining better knowledge promoting dietary diversity [48, 49]. A plausible explanation could be that mothers can gain more information about nutrition and childcare from mass media. Using the internet and television, mass media campaigns may improve nutrition, the inclusion of diverse food in the diet, as well as focus on water, sanitation, and hygiene practices that may affect a child’s growth and development.

Consistent with the previous research on dietary diversity [50], the present study also found that older children (18–23 months) had higher ADDI when compared to the younger age group (6–11 months). And in the past decade, the consumption of ADDI among both age groups has seen an increase of 21% in 2005–06 and 28.6% in 2015–16. The prominent reason could be the older government's child-focused support programs in the country, such as ICDS [51]. However, there is a continuous need for monitoring the progress of such mass-level schemes to address the issues of malnutrition in the country. The analysis also showed improved ADDI among children of higher birth order. In the sex of the child, between the years, the changes were seen (for the male child: 14.4% vs 20.3%; and for the female child: 13.9% vs 20.6%).

In the current study, the poorest wealth index contributed to low ADDI, whereas the wealthy household had the capacity to purchase diverse food items and consume a diversified diet. Similar results were observed in the pooled logistic regression analysis; the richest quintile had higher odds of ADDI among children than the poorest quintile. Other studies also reported a positive effect of wealth on increased intake of diversified diet among children in India [13, 52–54]. However, the differences in ADDI among children were decreased between the periods with a higher wealth index. Our study also highlighted children from SC and ST had higher odds of not receiving ADDI than those who belonged to other backward classes or other castes. However, there is limited literature comparing nutrition consumption among all social castes in India. At the same time, regional variations in intake of ADDI were also observed. Northeast and southern regions had low ADDI when compared with central and western areas [55]. A study based on the Consumption Expenditure survey also revealed similar findings; people in the northeast consumed the lowest quantity of legumes and processed goods [56]. Another study based on trend analysis from 1993 to 2012 delineated the different regional patterns of diverse diet consumption across India [57]. When we compared the urban and rural places of residence, the odds of ADDI among children were lower in rural residents despite the Anganwadi centres (AWC) that provide meals to preschool and school-going children in rural areas. Previous studies have documented that the centre workers are often stressed and overworked [58]. Lack of adequate infrastructure, delay in salaries, and insecurity of jobs were some of the reasons that impacted the motivation and performance of workers [59].

Previous studies have also reported low consumption of diverse dietary intake in specific caste, regions, and places of residence, indicating inequality among children [60, 61]. The Fairlie decomposition analysis [62] for ADDI in this study shows a wide gap between these two periods, 0.14 in 2005–06 and 0.20 in 2015–16.

To ensure adequate nutrition throughout complementary feeding could be a global health priority, however meeting the nutritionary needs of 6- to 24-month-old children is worth demanding [27], along with dietary quality rather than quantity often being the vital problem [63]. Undernutrition and adequate dietary deficiencies have been recurring problems in India. To make progress, efforts must focus on deep-rooted policy-level issues of undernutrition, examining how and why the problem persists in the system. To solve these issues, strengthening multifaceted strategies at the national level is the need of the hour. Hence, we suggest that the government should significantly focus on women’s development, empowerment, and increasing the resource base to access the right kind of nutrition essential for their children.

### Strength and limitations

The study is based on nationally representative data therefore the results can be generalized. The study used Infant and Young Child Feeding (IYCF) guidelines for estimating ADDI. The study has also some limitations. First, since the study used secondary data, the analysis was limited in order to use of those variables which were in data set. Hence, interpretations and inferences are drawn from our study might be limited in light of the included variables. Additionally, the NFHS employs cross-sectional designs, which restrict the analysis of causality on the noted outcomes. The key variables were self-reported as recall periods in 24 h by the mothers, and therefore, there is the likelihood of recall bias concerns. The study did not include the breastfeeding indicator for considering ADDI as there was huge missing cases.

## Conclusion

The study's findings reveal that there is a association between socioeconomic factors and ADDI among children and the trend is seen in nutritional consumption practices in India. Furthermore, our findings indicate that ADDI is consistently low among younger children, mothers with low education levels and no exposure to mass media, children of rural areas, poor households, and children from northeastern and southern India. Increasing awareness of the use of mass media, empowering women, and improving mothers’ education levels would be beneficial in the long term. Both media and education can facilitate mothers’ knowledge of feeding practices and dietary nutrition for infants and young children. The Indian government has worked on maternal and child health support programs; however, capacity-related challenges limit full coverage of infant and young children feeding interventions in India [64]. Overall, improving adequate dietary diversity requires a combination of strategies. Nutritional interventions will enhance the child’s health if the women’s resource base includes education and empowerment. Abridged Women’s Empowerment in Nutrition Index [65] that captures nutritional empowerment among women may help implement efforts to identify challenges women face and measure their autonomy and authorization in the house.

More research and analysis are needed across maternal and child improvement programs to intervene and sustain to deliver long-term efforts. Future, it is desired to examine the effect of the specific food groups across socio-economic groups included in dietary diversity on the nutritional status of children in India. We also recommend involving nutrition experts in collaborations and community programs could enhance and evaluate the benefits of programs. However, gaps should be reduced, and public health awareness and nutrition counselling programs should be formulated to promote ADDI with a household socio-economic focus.

## Supplementary Information

**Additional file 1:** **Table S1. **Pooled logistic regressionestimates for ADDI among children aged 6-23 months by their backgroundcharacteristics in India  

## Data Availability

The study utilizes secondary source of data which is freely available in public domain through https://www.iipsindia.ac.in/.

## Abbreviations

ADDI: Adequate diversified dietary intake
AOR: Adjusted odds ratio
CI: Confidence interval
ICDS: Integrated Child Development Services
MDD: Minimum Dietary Diversity
NFHS: National Family Health Survey
SC: Scheduled Class
ST: Scheduled Tribe
OBC: Other Backward Class
PCA: Principal component analysis
