# Supplementary material for: Contribution of socio-economic and demographic factors to the trend of adequate dietary diversity intake among children (6–23 months): evidence from a cross-sectional survey in India
Source: BMC Nutr. 2022 Dec 27;8:153. doi: 10.1186/s40795-022-00655-z (PMC9793661; doi:10.1186/s40795-022-00655-z)
Supplement: Supplementary file 1 — Additional file 1: Table S1. Pooled logistic regressionestimates for ADDI among children aged 6-23 months by their backgroundcharacteristics in India [file 40795_2022_655_MOESM1_ESM.docx]

| **Table-S1.** Pooled logistic regression estimates for ADDI among children aged 6-23 months by their background characteristics in India. | |
| --- | --- |
| **Background characteristics** | **AOR** |
|  | **95% CI** |
| **Year of survey** |  |
| 2005-06 | Ref. |
| 2015-16 | 1.29*(1.22,1.35) |
| **Mother's characteristics** |  |
| **Mother's age (in years)** |  |
| 15-24 | Ref. |
| 25-34 | 1.13*(1.08,1.17) |
| 35+ | 1.18*(1.09,1.27) |
| **Mother's educational status** |  |
| Not educated | Ref. |
| Primary | 1.11*(1.04,1.17) |
| Secondary | 1.37*(1.3,1.44) |
| Higher | 1.65*(1.53,1.78) |
| **Media exposure** |  |
| Exposed | Ref. |
| Not exposed | 1.29*(1.22,1.36) |
| **Child characteristics** |  |
| **Child's age (in months)** |  |
| 6-11 | Ref. |
| 12-17 | 2.97*(2.83,3.11) |
| 18-23 | 3.94*(3.76,4.13) |
| **Sex** |  |
| Male | Ref. |
| Female | 0.99(0.96,1.03) |
| **Birth order** |  |
| 1 | Ref. |
| 2 | 1.17*(1.12,1.22) |
| 3+ | 1.06*(1.01,1.12) |
| **Household characteristics** |  |
| **Wealth quintile** |  |
| Poorest | Ref. |
| Poorer | 1.10*(1.04,1.17) |
| Middle | 1.21*(1.13,1.29) |
| Richer | 1.32*(1.23,1.41) |
| Richest | 1.42*(1.31,1.54) |
| **Religion** |  |
| Hindu | Ref. |
| Muslim | 1.27*(1.21,1.34) |
| Christian | 1.37*(1.27,1.47) |
| Others | 1.09(1,1.19) |
| **Caste** |  |
| Scheduled Caste | Ref. |
| Scheduled Tribe | 1.11*(1.04,1.18) |
| Other Backward Class | 0.93*(0.88,0.98) |
| Others | 1.07*(1.01,1.13) |
| **Place of residence** |  |
| Urban | Ref. |
| Rural | 0.97(0.92,1.01) |
| **Regions** |  |
| North | Ref. |
| Central | 0.76*(0.72,0.8) |
| East | 1.28*(1.21,1.36) |
| North East | 1.90*(1.79,2.02) |
| West | 0.83*(0.77,0.9) |
| South | 1.89*(1.78,2.01) |

AOR: Adjusted odds Ratio; CI: Confidence interval; Ref: Reference; *if p<0.05
